# WWP1 targeting MUC1 for ubiquitin-mediated lysosomal degradation to suppress carcinogenesis

**DOI:** 10.1038/s41392-021-00660-x

**Published:** 2021-08-18

**Authors:** Chunhua Liao, Liping Yu, Zhi Pang, Huayun Deng, Xiaodong Liao, Shengze Li, Jinke Cheng, Min Qi, Guoqiang Chen, Lei Huang

**Affiliations:** 1 Department of Histoembryology, Genetics and Developmental Biology, Key Laboratory of Cell Differentiation and Apoptosis of Chinese Ministry of Education, Shanghai Key Laboratory of Reproductive Medicine, Shanghai Jiao Tong University School of Medicine, Innovative research team of high-level local universities in Shanghai, Shanghai, P. R. China; 2 Department of Plastic Surgery, Xiangya Hospital, Central South University, Changsha, P. R. China; 3 Liver Cancer Institute, Zhongshan Hospital, Key Laboratory of Carcinogenesis and Cancer Invasion, Ministry of Education, Fudan University, Shanghai, P. R. China

**Keywords:** Drug development, Oncogenes

**Dear Editor**,

Mucin 1 (MUC1) contains N- and C- subunit that forms a heterodimer on the apical surface of luminal epithelial cells. Nevertheless, MUC1 is aberrantly overexpressed in various cancers, such as breast cancer, lung cancer, and prostatic cancer, which critically contributes to tumorigenesis and poor clinical outcomes.^1^ Several strategies have been developed for targeted-inhibition of MUC1 including vaccines, monoclonal antibodies (MAb), and polypeptide. Vaccines usually recognize abnormal glycosylated regions in the VNTR of MUC1-N, MAb is mainly aimed at MUC1-N with little success, polypeptide inhibitors are applied to blocking the dimerization through CQC motif in MUC1-C, among which polypeptide inhibitor GO203 has entered clinical trials. However, none of the MUC1-targeted therapies are currently feasible in clinical treatment.^2^ The overexpression of MUC1 in cancer is partly due to the gene amplification and increased transcription. But less is known about how MUC1 protein is maintained. Deciphering the mechanism underlying MUC1 degradation will provide a novel strategy to reduce MUC1 abundance in cancer.

To discover novel regulators of MUC1, we performed LC–MS/MS assay and identified WW domain-containing ubiquitin E3 ligase 1 (WWP1) that interacts with MUC1 (Supplementary Table S1 and Fig. 1a). Protein levels of MUC1 and WWP1 were detected and present an inverse correlation in cell lines from breast cancer, liver cancer and non-small cell lung cancer^3^ (Supplementary Fig. S1a–c). Consistently, using complementary genetic approaches, we revealed that WWP1 negatively regulated MUC1 in protein level rather than mRNA level (Fig. 1b and Supplementary Fig. S1d–h). WWP1 reduced MUC1 abundance in a dose-dependent manner (Supplementary Fig. S1i). Overexpression of WWP1 significantly shortened the half-life of MUC1 protein (Fig. 1c and supplementary Fig. S1j). These results indicate that WWP1 triggers the turnover of MUC1 protein.

**Fig. 1:**
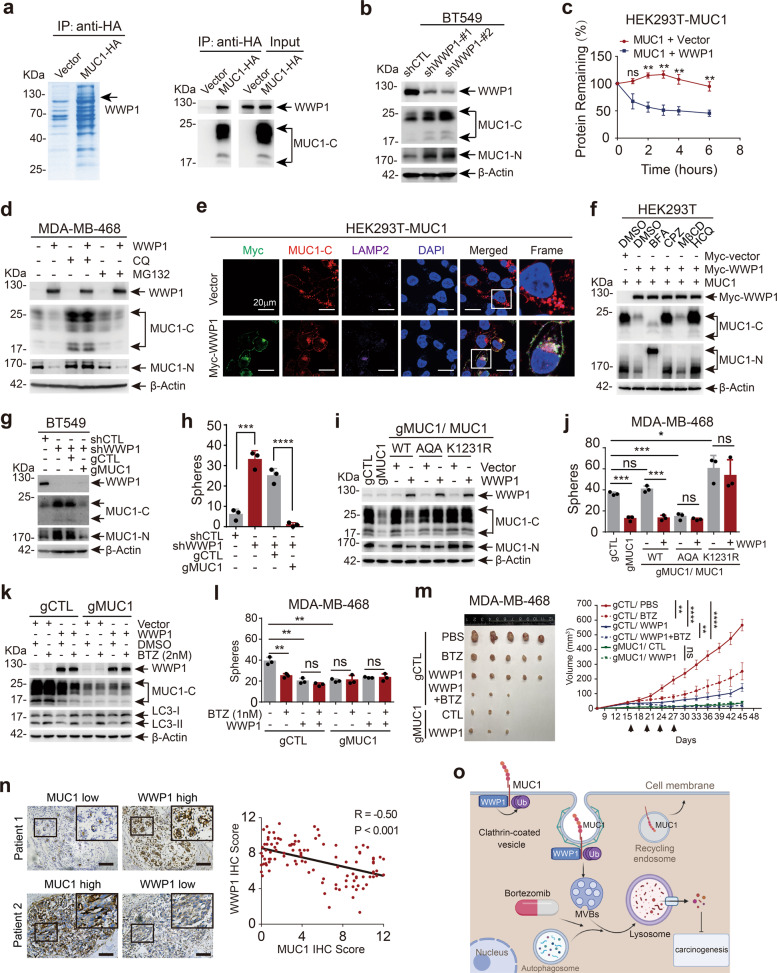
WWP1 triggers MUC1 degradation through ubiquitin-mediated lysosomal pathway to suppress carcinogenesis. **a** Mass spectrometry and immunoblot for proteins immunoprecipitated by HA-beads. HEK293T cells were transfected with Vector-HA and MUC1-HA. **b** Immunoblot for the indicated proteins in BT549/shCTL, BT549/shWWP1-#1, and BT549/shWWP1-#2 cells. **c** Immunoblot for MUC1 and WWP1 protein from HEK293T cells treated with CHX (30mg/ml) in absence or presence of Myc-WWP1 for 0–6 h. β-Actin was measured as the loading control. The percentage of remaining MUC1-C protein was calculated at each time point. **d** Immunoblot for the indicated proteins from MDA-MB-468/CTL and MDA-MB-468/WWP1 cells treated with 50 μM lysosome inhibitor CQ for 24 h or 10 μM proteasome inhibitor MG132 for 6 h, or DMSO as control. **e** Representative images of immunofluorescence (IF) staining of HA (red), LAMP2 (purple) together with Myc (green) in HEK293T cells transfected MUC1-HA with or without Myc-WWP1. DAPI (blue) was used to visualize nucleus. Scale bars: 20 μm. **f** Immunoblot for the indicated proteins from HEK293T cells co-transfected MUC1-HA with Myc-Vector or Myc-WWP1 treated with DMSO, Golgi inhibitor BFA (8 μM), clathrin-dependent endocytosis inhibitor CPZ (10 μM), cavolin-dependent endocytosis inhibitor MβCD (5 mM), or lysosome inhibitor HCQ (50 μM) for 24 h. **g**, **h** Constructed the stable knockdown of WWP1 or thereafter CRISPR cas9-MUC1 cell line in BT549 cells. Western blot (**g**) and secondary mammosphere formation assay (**h**) were performed in indicated cells. **i**, **j** Co-transfected WWP1 and MUC1-WT or MUC1-AQA/K1231R mutants in MDA-MB-468/gMUC1 cells. Western blot (**i**) and mammosphere formation assay (**j**) were performed in indicated cells. **k** Immunoblot for the indicated proteins from MDA-MB-468/gCTL and MDA-MB-468/gMUC1 cells transfected vector or WWP1 and treated with DMSO or bortezomib (BTZ, 2 nM) for 24 h. **l** Mammosphere formation assay in indicated cells treated with DMSO or bortezomib (1 nM). **m** MDA-MB-468/gCTL + Vector, MDA-MB-468/gCTL + WWP1, MDA-MB-468/gMUC1 + Vector, and MDA-MB-468/gMUC1 + WWP1 cells were subcutaneously injected into nude mice (5 × 10^6^ cells per mouse). When the size of the xenograft reached 4 mm × 4 mm, the mice (MDA-MB-468/gCTL + Vector and MDA-MB-468/gCTL + WWP1) were randomly alienated into two groups and treated intraperitoneally with bortezomib (0.25 mg/kg) twice a week for 2 weeks, the control group was treated with PBS. Tumor volumes were measured every 3 days. On day 45 after subcutaneous injection, tumors were harvested and photographed. **n** Representative images of IHC detection for MUC1 and WWP1 in 108 breast cancer specimens (left). Scale bars: 100 μm. Correlation between MUC1 and WWP1 expression in breast cancer specimens was plotted by Pearson correlation analysis (right). **o** A schematic diagram depicting a model for WWP1-mediated lysosome degradation of MUC1 in cancer. WWP1 interacts with MUC1 in cell membrane and promotes MUC1 degradation via clathrin-mediated endosome/lysosome pathway. WWP1-mediated MUC1 downregulation is associated with suppression of tumorigenesis. WWP1-mediated MUC1 degradation can be stimulated through activation of autophagy/lysosome by proteasome inhibitor bortezomib. The data in **c**, **m** presented as mean ± SEM. The data in **h**, **j**, **l** presented as mean ± SD of triplicates. ns means no significance, *p < 0.05, **p < 0.01, ***p < 0.001, ****p < 0.0001

Furthermore, WWP1-mediated degradation of MUC1 was considerably hindered by lysosome inhibitor chloroquine (CQ) rather than proteasome inhibitor MG132, suggesting WWP1-induced degradation of MUC1 through lysosome pathway (Fig. 1d and Supplementary Fig. S2a). This result was further corroborated by WWP1-induced co-localization of MUC1 and LAMP2 (Fig. 1e). Inhibitors were employed to examine the subcellular location where WWP1 degraded MUC1. The result validated that only CPZ (an inhibitor for clathrin-dependent endocytosis) prominently blocked WWP1-mediated MUC1 degradation (Fig. 1f), suggesting a crucial role of clathrin-dependent endocytosis. Immunofluorence staining revealed that WWP1 increased MUC1 level in early- and late- endosomes and reduced MUC1 in circulating endosomes (Supplementary Fig. S2b–d). Activation of autophagy by rapamycin augmented WWP1-dependent degradation of MUC1 in BT549 cell line (Supplementary Fig. S2e). Collectively, these results demonstrate that WWP1 degrades MUC1 through clathrin-mediated endosome/lysosome pathway.

A positive correlation of WWP1 level and growth inhibition was observed in all of 4 cell lines (Supplementary Fig. S3a–d). Ablation of MUC1 using CRISPR-Cas9 (gRNAs) in WWP1-silenced BT549 cells led to a diminished cell proliferation (Fig. 1g and Supplementary Fig. S3e), indicating that WWP1 targeted MUC1 for growth inhibition. Similar results were obtained in colony formation (Supplementary Fig. S3f), migration (Supplementary Fig. S3g, h) and mammospheres formation assay (Fig. 1h and Supplementary Fig. S3i). These results illustrate that WWP1 alleviates cell malignance through reduction of MUC1 level.

The WWP1C890A mutant lacking of E3 ligase activity had little, if any effect on MUC1 turnover (Supplementary Fig. S4a, b), indicating the E3 ligase activity is essential for WWP1 to degrade MUC1. Co-IP experiments with WWP1 truncation mutants demonstrated that WWP1 binds to MUC1 through its WW1/2 domains (Supplementary Fig. S4c, d). Accordingly, WWP1 wildtype but not C890A mutant ubiquitinates MUC1 (Supplementary Fig. S4e). K48- and K63-linked polyubiquitination are the canonical signals to flag substrate proteins for degradation, our result revealed that K63 linkage rather than K48 linkage was mainly responsible for MUC1 ubiquitination by WWP1 (Supplementary Fig. S4f). We then verified the potential ubiquitination sites of MUC1 by bringing mutations in possible lysine residues. Mutants in MUC1-C but not MUC1-N were refractory to WWP1-induced degradation. Notably, MUC1-K1231R was almost completely resistant to degradation by WWP1 (Supplementary Fig. S4g–j). These results suggest that WWP1 promotes K63-linked ubiquitination of MUC1 primarily at the K1231 residue.

The interaction of endogenous MUC1 and WWP1 was confirmed in BT549 cells (Supplementary Fig. S5a). We further mapped the motif in MUC1 required for the binding to WWP1. Interestingly, deletion of either of two potential PY motifs that WWP1 may recognizes^4^ had little, if any effect on WWP1-induced degradation of MUC1 (Supplementary Fig. S5b). Assessing on MUC1-CD truncation mutants displayed that WWP1 interacted with MUC1-CD1–20 (Supplementary Fig. S5c, d). Deletion of CD1–6 in MUC1 considerably attenuated WWP1-induced MUC1 degradation (Supplementary Fig. S5e). The MUC1-CQC was finally proved to be indispensable to interact with WWP1 (Supplementary Fig. S5f, g). Consistently, MUC1-AQA and MUC1-K1231R mutants that were resistant to ubiquitination by WWP1 cannot be degraded by WWP1 (Fig. 1i) and failed to rescue either cell proliferation (Supplementary Fig. S5h) or mammosphere formation (Fig. 1j) in MDA-MB-468/gMUC1 cells. Our results also simplify a necessity of membrane localization for MUC1 turnover by WWP1 (Supplementary Fig. S5i–m). These results clarify that WWP1 suppress cancer cell growth by promoting ubiquitination and degradation of MUC1.

Unlike the known substrates of WWP1 that are all degraded through the proteasome pathway,^4^ WWP1 directed MUC1 degradation through the lysosome pathway. Given that proteasome inhibitors can also induce anti-tumor effects through activating the autophagy-lysosome pathway,^5^ we hypothesized that the proteasome inhibitor might enhance WWP1-mediated lysosomal degradation of MUC1 via hampering WWP1 activity in the proteasome pathway. Indeed, bortezomib treatment significantly reduced MUC1 with WWP1 existent in both BT549 and MDA-MB-468 cells (Fig. 1k and Supplementary Fig. S6a). Management with bortezomib was associated with inhibition of cell proliferation and mammospheres formation in MDA-MB-468/gCTL rather than MDA-MB-468/gMUC1 cells (Fig. 1l and Supplementary Fig. S6b, c). Finally, we employed xenograft models to assess the anti-tumor activity of bortezomib under the condition of WWP1 overexpression or MUC1 ablation. Bortezomib treatment markedly delayed tumor growth in WWP1-expressing MDA-MB-468/gCTL but not in MDA-MB-468/gMUC1 cells without affecting mice body weight (Fig. 1m and Supplementary Fig. S6d, e). IHC staining assay on xenograft tissues confirmed that the expression of MUC1 was considerably decreased on the treatment of bortezomib or overexpression of WWP1 (Supplementary Fig. S6f). In summary, these results demonstrate that bortezomib prominently stimulates WWP1-induced MUC1 degradation and tumor destruction.

In order to test the clinical relevance for the connection between MUC1 and WWP1, we analyzed patient samples in cBioPortal database and found that gene amplification of MUC1 and WWP1 presents noteworthy mutual exclusiveness (Supplementary Fig. S7a–c), we further analyzed 108 human breast cancer samples and found a reverse correlation between the levels of MUC1 and WWP1 (Fig. 1n and Supplementary Fig. S7d). Furthermore, out of 17 specimens that had both high expression of MUC1 and WWP1, 14 of them (82.4%) displayed different distribution of MUC1 and WWP1 in tumor cells (Supplementary Fig. S7e). Collectively, these results show an inverse association of MUC1 level with WWP1 expression in cancer tissues.

The MUC1 overexpressed on cell membrane involves in multiple oncogenic signaling pathways (for example EGFR and β-catenin, etc.)^2^ and promotes tumorigenesis. Our study reveals that WWP1 can degrade the membrane MUC1 through the lysosomal pathway, thereby inhibiting tumor growth. Importantly, we validate the function of proteasomal inhibitor-bortezomib in promoting WWP1-mediated MUC1 degradation, providing a novel strategy for management of WWP1/MUC1 positive cancers (Fig. 1o).

## Supplementary information

WWP1 targeting MUC1 for ubiquitin-mediated lysosomal degradation to suppress carcinogenesis

## Data Availability

All the data used for the current study are available from the corresponding author upon reasonable request.
