# Supplementary material for: WWP1 targeting MUC1 for ubiquitin-mediated lysosomal degradation to suppress carcinogenesis
Source: Signal Transduct Target Ther. 2021 Aug 18;6:297. doi: 10.1038/s41392-021-00660-x (PMC8371114; doi:10.1038/s41392-021-00660-x)
Supplement: Supplementary file 1 — WWP1 targeting MUC1 for ubiquitin-mediated lysosomal degradation to suppress carcinogenesis [file 41392_2021_660_MOESM1_ESM.docx]

Supplementary Materials for

WWP1 targeting MUC1 for ubiquitin-mediated lysosomal degradation to suppress carcinogenesis

Chunhua Liao^1^, Liping Yu^2^, Zhi Pang^1,3^, Huayun Deng^1^, Xiaodong Liao^1^, Shengze Li^1^, Jinke Cheng^1^, Min Qi^2*^, Guoqiang Chen^1*^, Lei Huang^1*^

^1^Department of Histoembryology, Genetics and Developmental Biology, Key Laboratory of Cell Differentiation and Apoptosis of Chinese Ministry of Education, Shanghai Key Laboratory of Reproductive Medicine, Shanghai Jiao Tong University School of Medicine, Innovative research team of high-level local universities in Shanghai, Shanghai, P. R. China

^2^Department of Plastic Surgery, Xiangya Hospital, Central South University, Changsha, P. R. China.

^3^Liver Cancer Institute, Zhongshan Hospital, Key Laboratory of Carcinogenesis and Cancer Invasion, Ministry of Education, Fudan University, 180 Fenglin Road, Shanghai, P. R. China

* Correspondence to: Lei Huang (Email: [leihuang@shsmu.edu.cn](mailto:leihuang@shsmu.edu.cn)), Guoqiang Chen (Email: [chengq@shsmu.edu.cn](mailto:chengq@shsmu.edu.cn)) or Min Qi (Email: qimin05@163.com).

**This file includes:**

Materials and Methods

Abbreviations

Figures. S1 to S7

Supplementary Table 1 to 2

References for supplementary materials

**Materials and methods**

Cell lines and cell culture

The human breast cancer BT549，human liver cancer Bel-7402，human non-small cell lung cancer NCI-H1975，and human embryonic kidney 293T were purchased from the Cell Bank of the Chinese Academy of Sciences in Shanghai. The human breast cancer MDA-MB-468 was purchased form American Type Culture Collection (ATCC). HEK293T and Bel-7402 cells were cultured in DMEM containing 10% fetal bovine serum (Gibco, Grand Island, NY, USA) in a 5% CO_2_ incubator at 37℃. BT549 and NCI-H1975 cells were maintained in RPMI1640 supplemented with 10% FBS in a 5% CO_2_ incubator at 37℃. MDA-MB-468 cells were cultured in L15 supplemented with 10% FBS in non-CO_2_ incubator at 37℃. All cell lines authentication were performed by using short tandem repeat profiling and no signs of mycoplasma contamination.

Plasmids and transfection

HEK293T cells were transfected with plasmids using Nano293T Transfection Reagent (NCM, China) according to the manufacturer’s instructions. Viral supernatant was harvested at 48 h post-transfection through a 0.45-μm filter.

WWP1 overexpression cells were established by infecting with virus vector of plvx-IRES-puro/WWP1. WWP1 knock down cells were established by the WWP1-specific shRNA sequences (shCTL: 5’-CTCGCTTGGGCGAGAGTAA-3’; shWWP1-#1: 5’-AGGTACTTTGTTGATCATA-3’; shWWP1-#2: 5’-ACAAGAACAACAACATTCA-3’). MUC1 deficient cells was established using a CRISPR/Cas9 system, which was described previously^1^. The stable transfected cell lines were then screened by puromycin.

Drugs and antibodies

The following drugs and antibodies were used in our experiments: CQ (Sigma-Aldrich, St. Louis, MO, USA), MG132, BFA, CPZ, MβCD, HCQ and bortezomib (TargetMol, China), rapamycin (Selleck Chemicals, Houston, TX, USA), anti-MUC1-C antibody (Cell Signaling Technology, Danvers, MA, USA), anti-MUC1-N antibody (Invitrogen, Carlsbad, CA, USA), anti-WWP1 antibody (Abnova, USA), anti-HA antibody (Proteintech Group, Chicago, USA), anti-myc antibody (Santa Cruz, CA, USA), anti-Ub antibody (Santa Cruz, CA, USA), anti-LAMP2 antibody (Proteintech Group, Chicago, USA), anti-LC3 antibody (Proteintech Group, Chicago, USA), anti-p62 antibody (Proteintech Group, Chicago, USA) and anti-β-actin antibody (Merck Millipore Billerica, MA, USA).

Mass spectrometry

MUC1-HA (pIRESPuro2-MUC1-HA), or Vector-HA (pIRESPuro2-Vector-HA) as a control, was transiently expressed in HEK293T cells and immunoprecipitated using HA beads (Santa Cruz, CA, USA) in NETN150 buffer with protease inhibitor cocktails and phosphatase inhibitor cocktails (Sigma-Aldrich). Beads were washed five times. Immunoprecipitation proteins were separated by SDS-PAGE and stained with coomassie blue. The band excised from the gel was subjected to reduction, carbamidomethylation, and tryptic digestion. Peptide sequences were determined by mass spectrometry using an Orbitrap Fusion LUMOS mass spectrometer (Thermo Fisher Scientific) connected to an Easy-nLC 1200 via an Easy Spray (Thermo Fisher Scientific).

Quantitative real-time PCR (RT-qPCR)

Total RNAs were extracted from cultured cells using Trizol reagent (Invitrogen, Carlsbad, CA) as instructed. cDNA was synthesized with the use of M-MLV Reverse Transcriptase synthesis kit (Promega Madison, WI, USA). RT–PCR was performed with SYBR Green PCR Master mix kit (Applied Biosystems, Warrington, UK) in accordance with the manufacturer’s instructions on the ABI Prism 7900 HT Fast real-time PCR system. The expression of each target mRNA was based on the cycle threshold (Ct). Experiments were repeated for three times. The primer sequences used are listed in Supplementary Table 2.

Western blot

Cells were harvested and lysed directly in NETN150 buffer (NaCl 150 mM, EDTA 1 mM，Tris 20 mM pH 7.6, NP40 0.5%)，with protease inhibitor cocktails (Sigma-Aldrich). The samples were quantified，then denatured, boiled, and separated by SDS-PAGE. Proteins were transferred to PVDF membranes and blocked with 5% nonfat milk. The membrane was incubated with primary antibodies overnight thereafter incubated with HRP-linked secondary antibodies. Chemiluminescence detection with ECL.

Co-Immunoprecipitation

Protein samples were washed with PBS then harvested and lysed directly in NETN150 buffer（with 1% of NP40）. Lysates were incubated with HA-beads /myc-beads (Santa cruz, USA) with rotation at 4℃ after quantified. Beads were washed by NETN150 lysis buffer for 3 times. Proteins were released form beads after denatured and boiled, then separated with SDS-PAGE for immunoblotting.

GST pull-down assay

The pSUMO-WWP1 plasmid was transformed into BL21（DE3）competent cells. The recombinant SUMO-WWP1 proteins were expressed by IPTG induction for 18h at 16°C. GST beads with MUC1-CD or -AQA proteins were incubated with recombinant WWP1 for 12h at 4°C, followed by washing 5 times with NETN150 buffer. Complex proteins were subjected to immunoblot analyses.

Immunofluorescence

Cells were plated on coverslips and cultured for 24h, washed by PBS thereafter fixed with paraformaldehyde for 10min, treat with Triton X-100 for 10 min and blocked with 5% goat serum (Gibco, Grand Island, NY, USA) for 1h at room temperature. primary antibodies were incubated with 5% goat serum at 4℃, overnight. The cells were washed in PBS for three times and incubated with fluorescent conjugated secondary antibodies for 2 h at 37℃. After washing three times in PBS, the coverslips were covered on the slides with DAPI (Sigma, Saint Louis, MO, USA). The images of cells were captured with Confocal Microscopes (Nikon, Tokyo, Japan).

Cell viability assay

Cells were planted in 96-well plates. Cell viability was measured using the cell counting kit 8 (CCK8) according to the manufacturer’s protocol (NCM, China). After incubating with CCK8 reagent for 2h at 37℃，the absorbance at 450 nm was determined by Multiscan Spectrum (Thermo) at the same time every day. The experiment was repeated three times, each with three pairs of holes.

Colony formation assay

Cells (1000 cells/well) were planted in 6-well plates. The medium was changed every three days and stopped when visible colonies formed after nine days. After washing with PBS, cells were fixed in methanol for 15min and subject to staining with crystal violet and photograph. The experiment was repeated three times, each with three pairs of holes.

Sphere formation assay

Cells were suspended in DMEM/F12 serum-free medium, which was described previously^1^ and cultured in ultra-low attachment plates (Corning, Corning, NY) for 5 days afterwards counted sphere numbers in each well. Harvest all of the cells, digested with TrypLE Express (Gibco, Grand Island, NY, USA) and washed twice with PBS. Cells then were subsequently resuspended in ultra-low attachment plates for 2 weeks for analysis their secondary sphere-forming capacity.

Invasion assay

Transwell chamber was placed in a 24-well plate, 600 ul complete medium which contained 10% FBS was added to the lower chamber; cells (2x10^4^ cells/well) were added into the upper chamber with 200 ul serum-free medium and cultured at 37℃ with 5% CO2 for 24h. The invaded cells were fixated with methanol and stained with Crystal Violet. Photos were taken from different fields under 10-fold microscope to count the number of migrated cells and make statistical analysis. The results were quantitated in 3 independent experiments.

Wound healing assay

After cell reached 100% confluence, scratched cells with a 200-μl pipette tip. Discard the medium, remove the floating cells by washing with PBS for 3 times, then add serum-free medium. Migration photos were captured at 0 and 48 hours after scratching under a 10-fold microscope. Cell mobility was calculated as = (0h width -48h width) /0h width x100%. The results were quantitated in 3 independent experiments.

Animal studies

The xenograft tumor experiments were performed in agreement with the Shanghai Medical Experimental Animal Care Guidelines. Research was permitted by the Institutional Animal Care and Use Committee of Shanghai Jiao Tong University School of Medicine. The MDA-MB-468 cells (5x10^6^/100ul PBS) were subcutaneously injected into the right flank of 6-week-old female BALB/c nude mice. Once the size of the xenograft reached 4 mm x 4mm，the mice were randomly divided into two groups and treated intraperitoneally with bortezomib at a dose of 0.25mg/kg twice a week for 2 weeks, the control group was treated with an equivalent volume of PBS. Investigators were blinded to the group allocation. Tumor size was monitored by caliper ruler every 3 days. The volume was calculated using the formula: V = (length x width^2^)/2. At the end of treatment, the animals were euthanasia, and the tumors were isolated and weighed for immunohistochemical staining or western blot studies.

Public database

The breast cancer genomics data sets, the non-small lung cancer genomics data sets, and the liver cancer genomics data sets, were analysized genetic alterations (includes amplification, mutation, fusion, deletion) of MUC1 and WWP1 within the cBioPortal database^2,3^ (http://www.cbioportal.org/).

Human breast cancer specimens and IHC

Human breast cancer patients who underwent breast cancer surgery from October 2010 to May 2015 were obtained with informed consent from the Xiangya Hospital of Central South University. Slides were reviewed and blocks were identified based on the presence of adequate tumors and the representative nature of the overall tumor. The age of the specimens was 22-74 years old, all cases were confirmed by pathology after operation.

The paraffin sections were dewaxed and hydrated, and then endogenous peroxidase activity was blocked using 3% hydrogen peroxide. The mouse anti-human monoclonal WWP1 primary antibody (Abnova, America) was used at a dilution of 1:200, the rabbit anti-human monoclonal MUC1 primary antibody (CST, America) at 1:200 for overnight at 4℃. After incubation with the secondary antibody and a streptavidin enzyme conjugate, the complex was then visualized by development of the color with DAB and the slides were counterstained with hematoxylin.

Immunohistochemical staining was blindly scored as low or high gives a range of 0-12 according to the positive cell proportion (0: ≤5%, 1: <25%, 2: <50%, 3: <75%, and 4: ≥75%) and the staining intensity (0: negative, 1: weak, 2: intermediate, and 3: strong) for analysis. This study was compliant with the related ethical regulations regarding research involving human participants.

Statistical analysis

The statistical analyses were performed by GraphPad Prism version 6 software. The experiments used unpaired Student’s t-test. All data are presented as means ± SD and represent three independent experiments. ns stand for not significant; **p* < 0.05; ***p* < 0.01; ****p* < 0.001; and *****p* < 0.0001.

**Abbreviations**

| MUC1 | Mucin 1 |
| --- | --- |
| MUC1-C | C-terminal of MUC1 |
| MUC1-N | N-terminal of MUC1 |
| MUC1-CD | The cytoplasmic domain of MUC1 |
| VNTR | Variable number of tandem repeats |
| WWP1 | WW domain-containing ubiquitin E3 ligase 1 |
| LC–MS/MS | Liquid chromatograph-mass spectrometer |
| shRNA | Short hairpin RNA |
| RT-qPCR | Quantitative real time polymerase chain reaction |
| WB | Western blot |
| Co-IP | Co-immunoprecipitation |
| CQ | [Chloroquine](https://www.sigmaaldrich.com/catalog/product/usp/1118000?lang=zh&region=CN) |
| HCQ | Hydroxychloroquine sulfate |
| BFA | Brefeldin A |
| CPZ | Chlorpromazine hydrochloride |
| MβCD | Methyl-β-cyclodextrin |
| LAMP2 | Lysosomal-associated membrane protein 2 |
| LC3 | Microtubule associated protein 1 light chain 3 alpha |
| IHC | Immunohistochemical staining |

**Figure. S1**

**
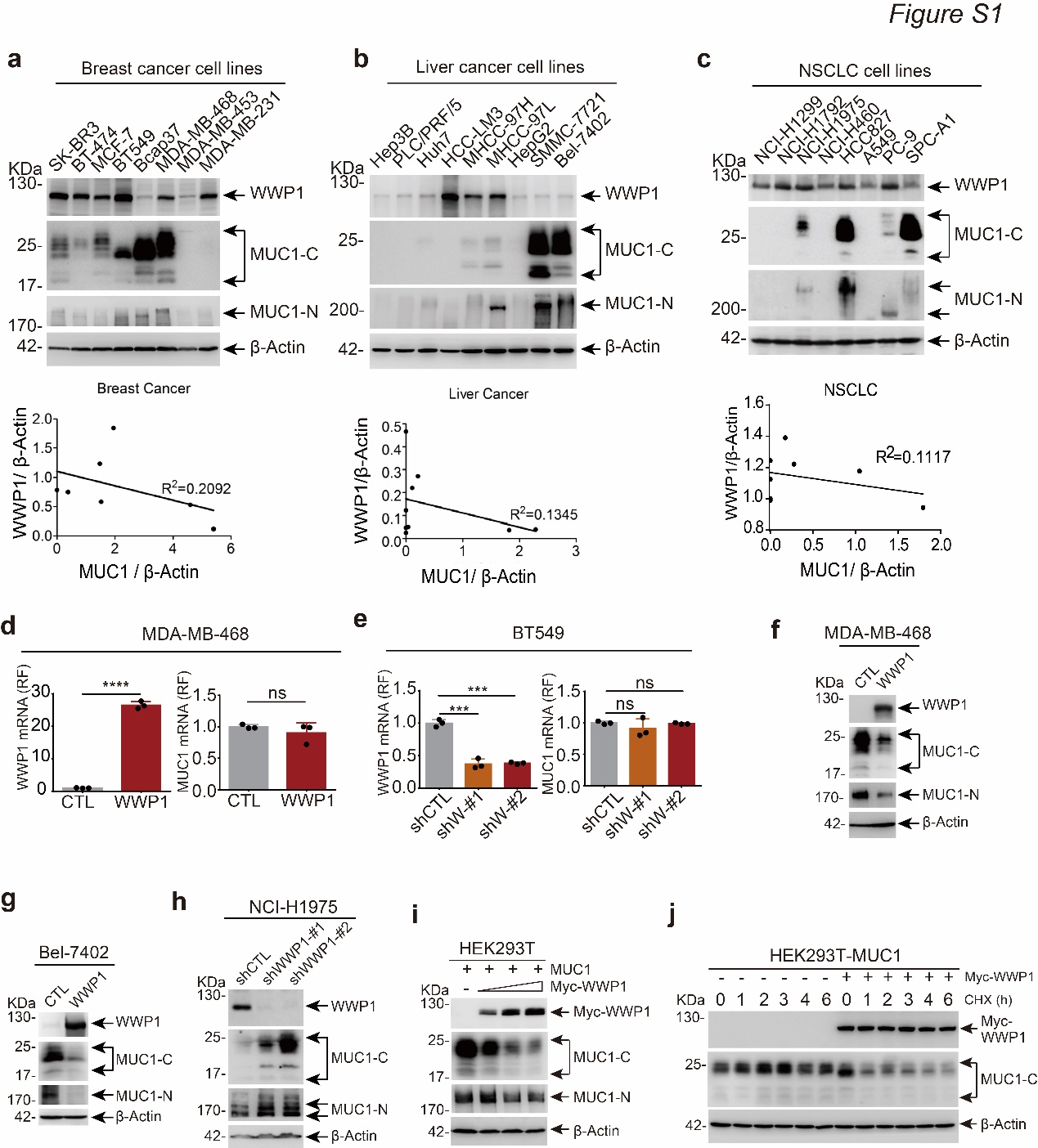
**

**a-c** Western blots were performed to detected the protein level of WWP1 and MUC1 in breast cancer (**a**), liver cancer (**b**) and non-small cell lung cancer (**c**) cell lines. β-Actin was measured as the control, quantification of relative WWP1 and MUC1-C levels in breast cancer, liver cancer and non-small cell lung cancer cell lines are presented below. **d** RT-qPCR were performed in MDA-MB-468/CTL and MDA-MB-468/WWP1 cells to detect indicated mRNAs. **e** RT-qPCR were performed in BT549/shCTL, BT549/shWWP1-#1 and BT549/shWWP1-#2 cells to detect indicated mRNAs. The relative fold (RF) of mRNA was calculated by normalizing with GAPDH. **f** Immunoblot for the indicated proteins in MDA-MB-468/CTL and MDA-MB-468/WWP1 cells. **g** Immunoblot for the indicated proteins in Bel-7402/CTL and Bel-7402/WWP1 cells. **h** Immunoblot for the indicated proteins in NCI-H1975/shCTL, NCI-H1975/shWWP1-#1 and NCI-H1975/shWWP1-#2 cells. **i** Immunoblot for the indicated proteins from HEK293T cells co-transfected with MUC1-HA and gradient concentration of Myc-WWP1. **j** Immunoblot for the indicated proteins from HEK293T cells transfected MUC1-HA with Myc-Vector and Myc-WWP1, and treated with CHX (30mg/ml) for 0-6h before collecting samples. The experiments shown in **d-j** were repeated three times, and the results of one representative experiment are shown. The data in **d, e** are presented as mean ± SD of triplicates. ns means no significance, ****p*<0.001, *****p*<0.0001.

**Figure. S2**


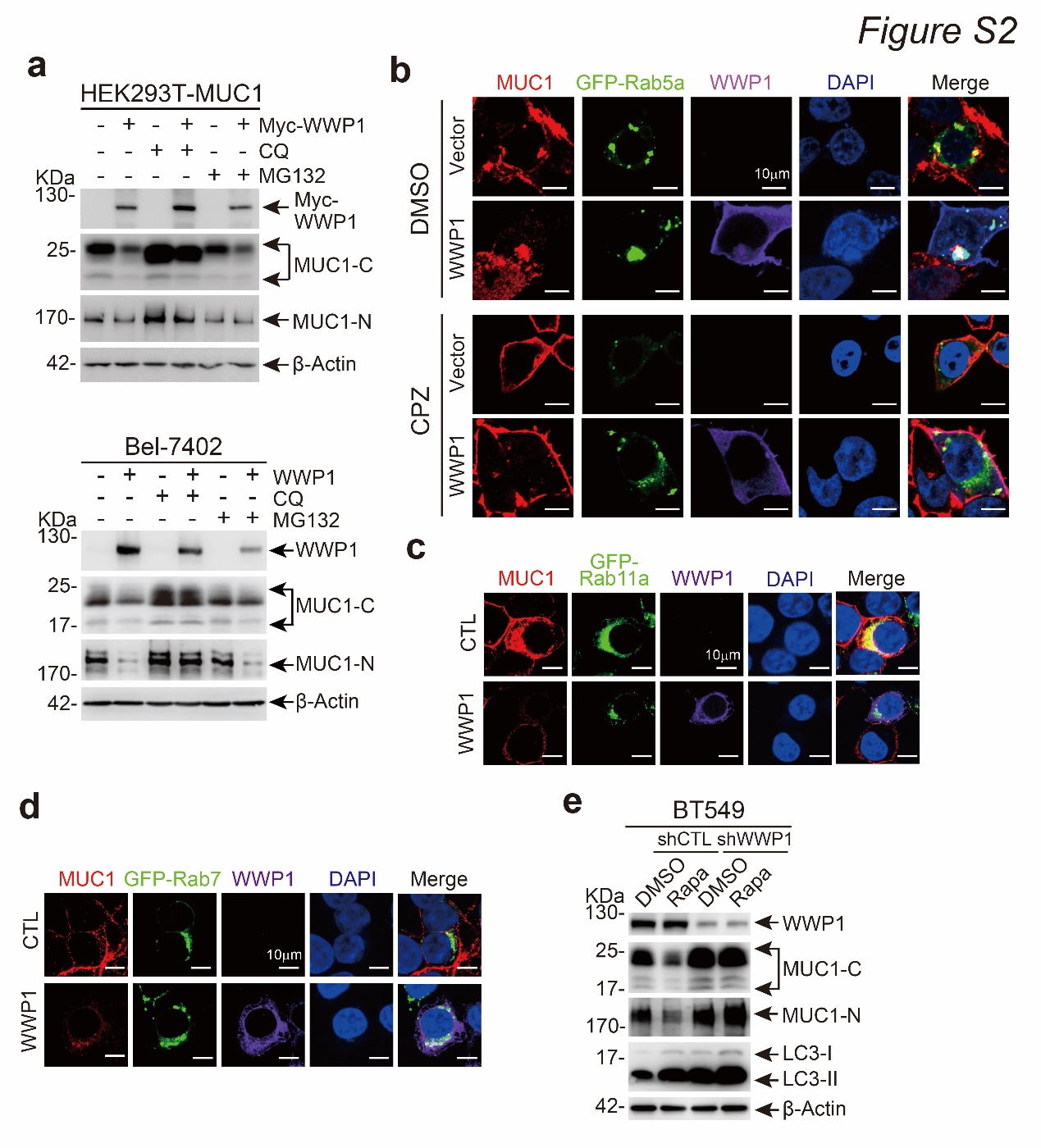


**a** Immunoblot for the indicated proteins from HEK293T cells co-transfected with MUC1-HA and Myc-WWP1, with the treatment in absence or presence of 100μM CQ for 24h or 20μM MG132 for 6h (upper). Immunoblot for the indicated proteins from Bel-7402/CTL and Bel-7402/WWP1 cells treated in absence or presence of 50μM CQ for 24h or 10μM MG132 for 6h (bottom). **b** Representative images of IF staining of HA (red), GFP-Rab5a (marker for early endosome) and Myc (purple) in HEK293T-MUC1-HA cells transfected Myc-Vector or Myc-WWP1, and treated with DMSO or CPZ (10ug/ml) for 30min. **c, d** Representative images of IF staining of HA (red), GFP-Rab11a (marker for circulating endosome) (**c**)/ GFP-Rab7 (marker for late endosome) (**d**) and Myc (purple) in HEK293T-MUC1-HA cells transfected Myc-Vector or Myc-WWP1. DAPI (blue) was used to visualize nuclei. Bars: 10μm. **e** Immunoblot for the indicated proteins from BT549/shCTL and BT549/shWWP1-#1 cells treated with DMSO or rapamycin (500nM) for 24h. The experiments shown in **a-e** were repeated three times, and the results of one representative experiment are shown.

**Figure. S3**


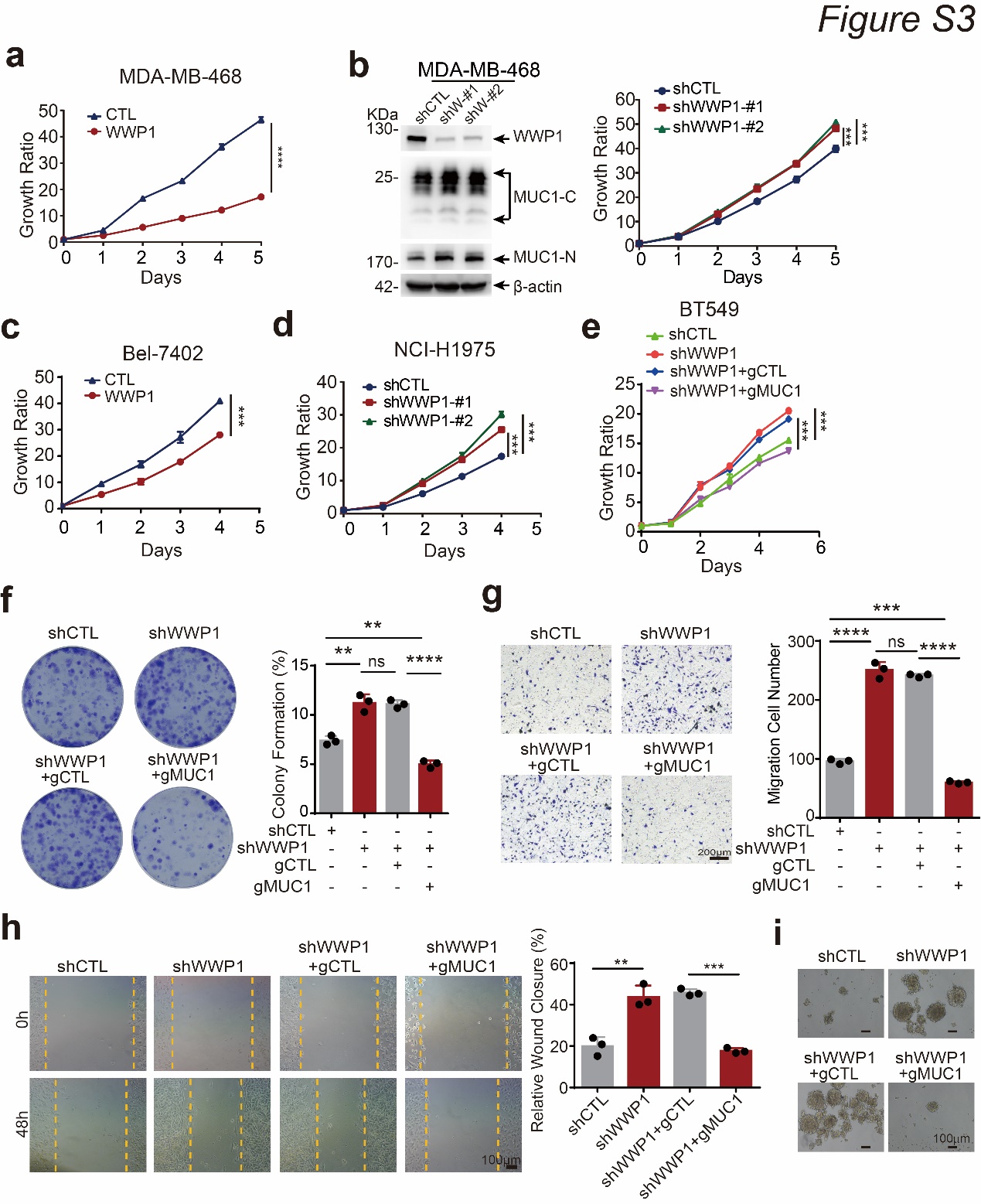


**a** Cell viability assay was analyzed for the stable expression of WWP1 or control in MDA-MB-468 cells. **b** Immunoblot for the indicated proteins from MDA-MB-468/shCTL, MDA-MB-468/shWWP1-#1, MDA-MB-468/shWWP1-#2 cells (left). Cell viability assay was analyzed (right). **c** Cell viability assay was analyzed for the stable expression of WWP1 or control in Bel-7402 cells. **d** Cell viability assay was analyzed for the stable knock down of WWP1 or control in NCI-H1975 cells. **e-i** Cell viability assay (**e**), colony formation (**f**), invasion (**g**), wound healing (**h**) and secondary mammosphere formation (**i**) was performed in indicated cells. The number of clones, cells or mammospheres was calculated in each hole. The data in panel **a-i** are presented as mean ± SD of triplicates. ns means no significance, ***p*<0.01, ****p*<0.001, *****p*<0.0001.

**Figure. S4**


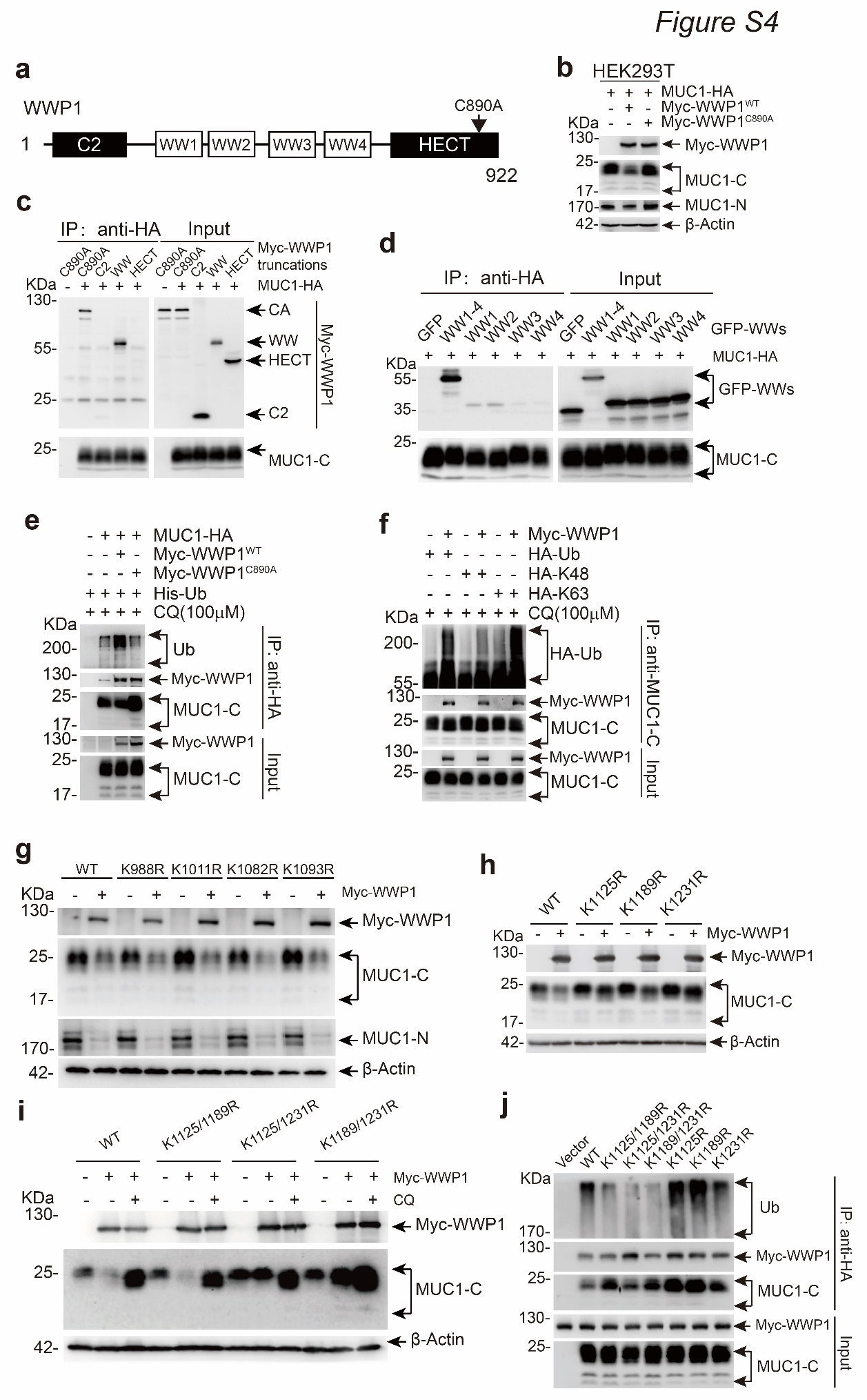


**a** A schematic of WWP1 protein which included C2, WW1/2/3/4 and HECT domain. **b** Immunoblot for the indicated proteins from HEK293T cells co-transfected with MUC1-HA together with Myc-WWP1 or Myc-WWP1C890A. **c** Co-IP of MUC1-HA and myc-tagged WWP1 truncations in HEK293T cells. **d** Co-IP of MUC1-HA and GFP-tagged WWP1-WW truncations in HEK293T cells. **e** HEK293T cells transfected with the indicated plasmids were treated with CQ (100μM) for 24h, and submitted to *in vivo* ubiquitination assay. **f** HEK293T cells co-transfected with MUC1, Myc-WWP1, HA-Ub (Ub WT or K48, K63) were treated with CQ (100μM) for 24h, and submitted to *in vivo* ubiquitination assay. **g-h** Immunoblot for the indicated proteins from HEK293T cells co-transfected Myc-WWP1 with MUC1-HA or mutants. **i** Immunoblot for the indicated proteins from HEK293T cells transfected Myc-WWP1 with MUC1-HA or various lysine-to-arginine mutants treated with DMSO or CQ (100μM) for 24h. **j** HEK293T cells co-transfected with Myc-WWP1, His-Ub together with MUC1-HA or HA-tagged various lysine-to-arginine mutants were treated with CQ (100μM) for 24h, and subjected to *in vivo* ubiquitination assay. The experiments shown in **b-j** were repeated three times, and the results of one representative experiment are shown.

**Figure. S5**


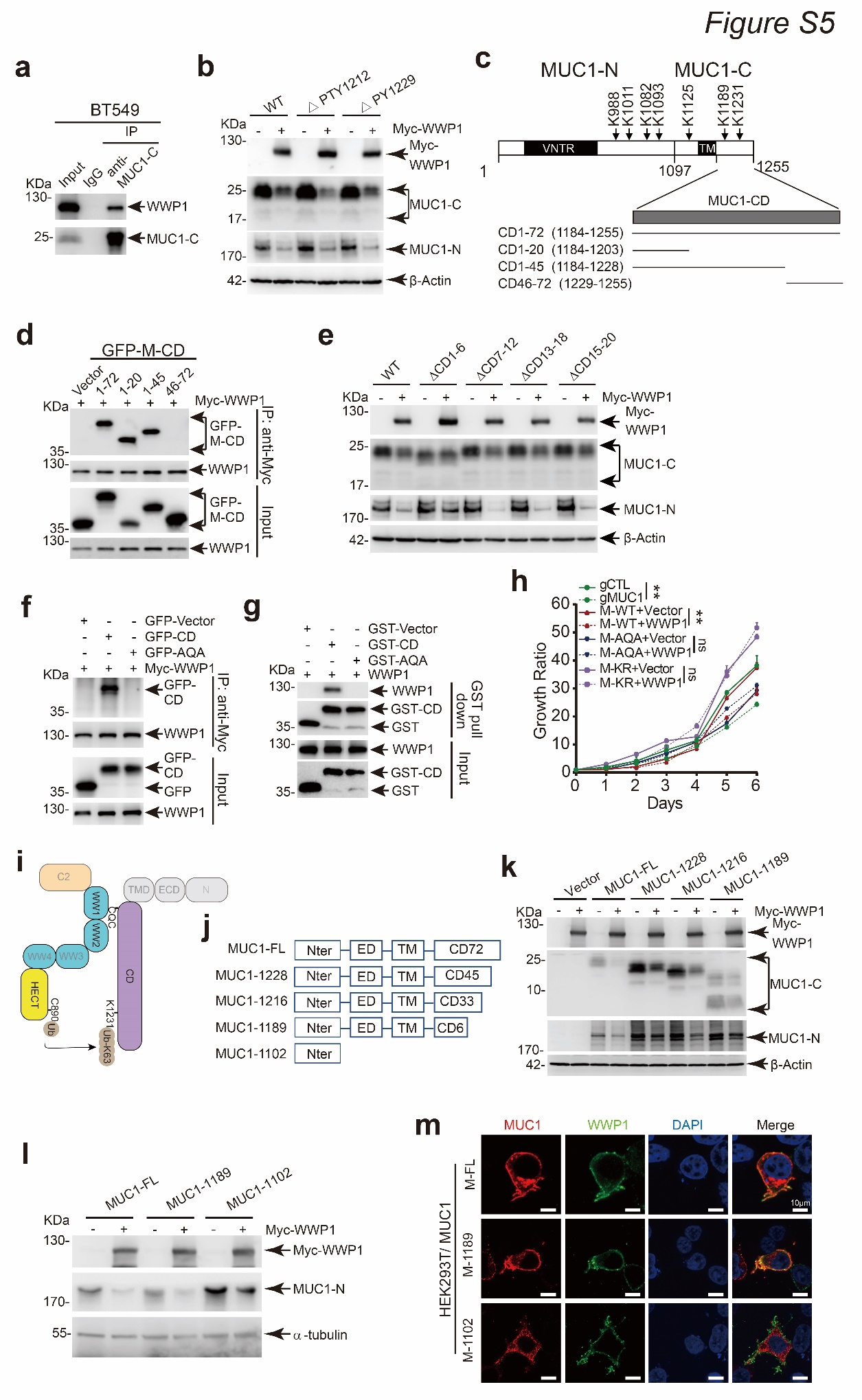


**a** Co-IP of endogenous MUC1 and WWP1 in BT549 cells. Assessed co-IP with IgG or anti-MUC1-C, the immunoprecipitates were analyzed with anti-WWP1. **b** Immunoblot for the indicated proteins from HEK293T cells transfected MUC1-HA, MUC1-△PTY_1212_-HA or MUC1-△PTY_1219_-HA with Myc-WWP1 for 48h. **c** Graphical model of MUC1 with lysine residues and MUC1-CD truncations. **d** Co-IP with anti-Myc antibody in HEK293T cells expressing EGFP-vector or EGFP-CD variants and Myc-WWP1. **e** Immunoblot for the indicated proteins from HEK293T cells co-transfected MUC1-HA or variant MUC1-CD deletion plasmids together with or without Myc-WWP1. **f** Co-IP with anti-Myc antibody in HEK293T cells expressing GFP-MUC1-CD or GFP-MUC1-CD (AQA) and Myc-WWP1. **g** Purified recombinant GST, GST-MUC1-CD or GST-MUC1-CD (AQA) beads was incubated with SUMO-WWP1. Immunoblot for the precipitated beads with indicated antibodies. **h** Co-transfected WWP1 and MUC1-WT or MUC1-AQA/K1231R mutants in MDA-MB-468/gMUC1 cells. Cell viability assay were performed in indicated cells. **i** Scheme illustrating how the WWP1 E3 ligase results in MUC1 ubiquitination and degradation. **j** A schematic of MUC1 truncations. **k** Immunoblot for the indicated proteins from HEK293T cells transfected Myc-WWP1 with MUC1-HA or indicated truncations. **l** Immunoblot for the indicated proteins from HEK293T cells transfected Myc-WWP1 with MUC1-HA, MUC1-1189 or MUC1-1102. **m** Representative images of IF staining of MUC1 (red) and Myc (green) in HEK293T cells co-transfected MUC1-HA, MUC1-1189 or MUC1-1102 plasmids with Myc-WWP1 for 24h. Bars: 10μm. The experiments shown in **a, b, d-h, k-m** were repeated three times, and the results of one representative experiment are shown.

**Figure. S6**

**
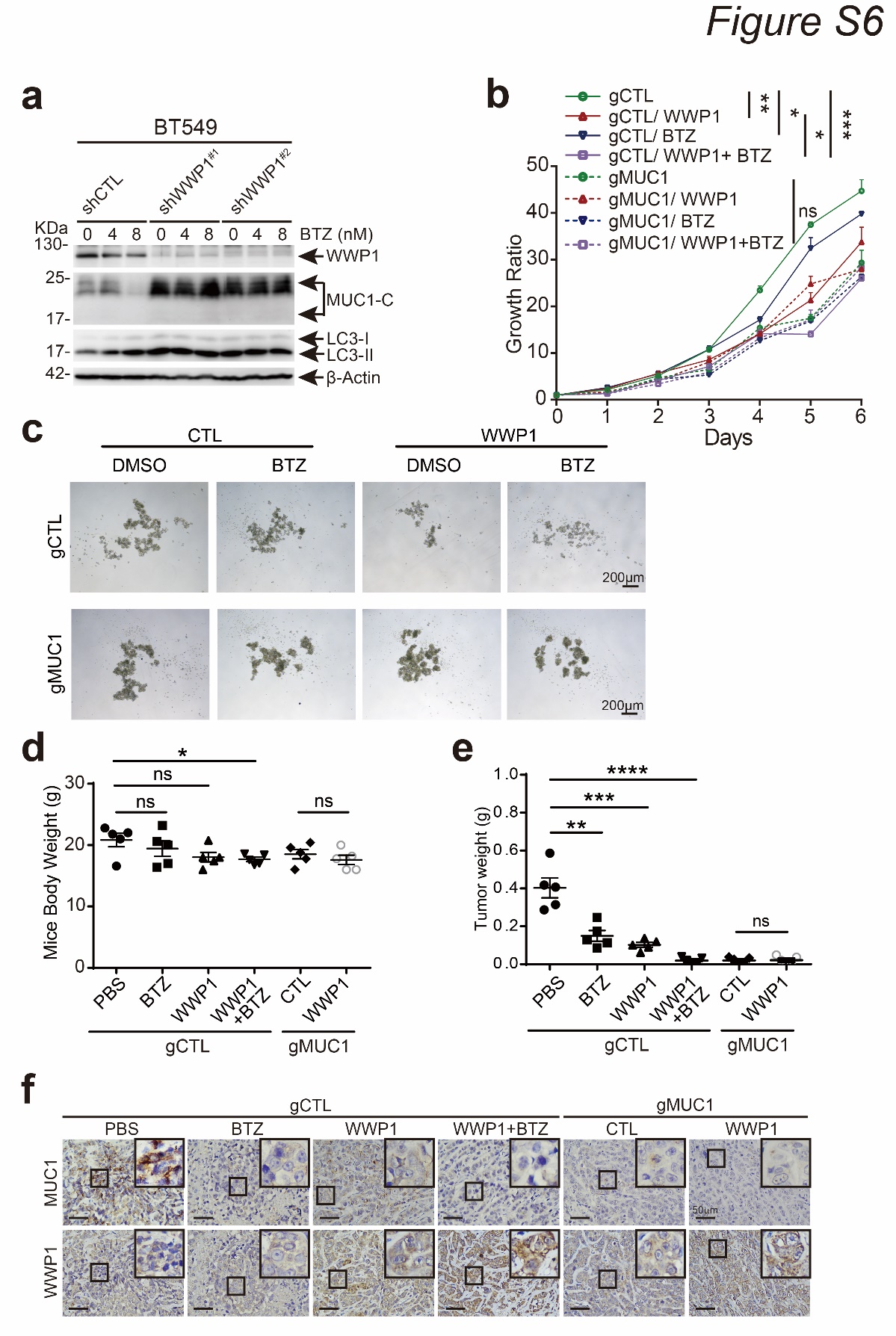
**

**a** Immunoblot for the indicated proteins from BT549/shCTL and BT549/shWWP1 cells treated with DMSO or Bortezomib (4nM and 8nM) for 24h. **b** Cell viability assay in indicated cells treated with DMSO or bortezomib (1nM). **c** Mammospheres formation assay in indicated cells treated with DMSO or bortezomib (1nM) for 5 days. Scale bar: 200μm. **d, e** Mice body weight (**d**) and the tumor weight (**e**) from indicated xenograft mice were tested (mean ± SEM, n=5). **f** Representative images of immunohistochemical (IHC) staining analysis of MUC1 and WWP1 in xenograft tissues. Scale bars: 50μm. The experiment shown in **a-c** was repeated three times, and the results of one representative experiment are shown.

**Figure. S7**


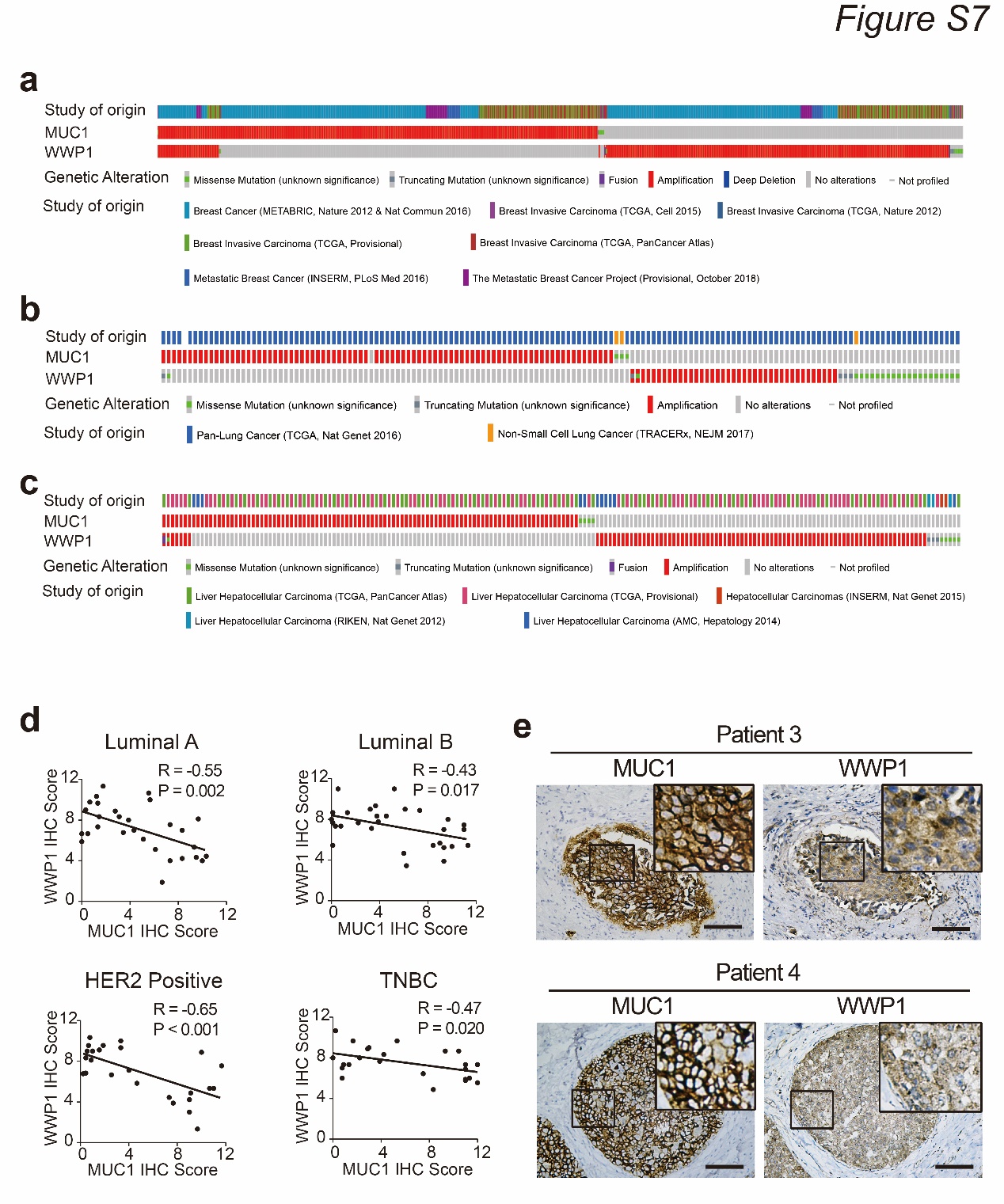


**a-c** Altered genetic alternations of MUC1/WWP1 in breast cancer, lung cancer, liver cancer patients in large-scale cancer genomics data sets within the cBioPortal database. Those cohort of breast cancer/lung cancer/liver cancer carrying both MUC1 and WWP1 truncating mutation, missense mutation, fusion, deletion or amplification. **d** Correlation between MUC1 and WWP1 expression in Luminal A/ Luminal B/ HER2 positive/ TNBC specimens was plotted by Pearson correlation analysis. **e** Immunostaining patterns for specimens harboring high expressed MUC1 and WWP1. Scale bars: 100μm.

| **Supplementary Table 1: MUC1 interacting proteins** | |  |
| --- | --- | --- |
| Accession Number | Identified Proteins | Molecular Weight (KDa) |
| sp\|P09874\|PARP1_HUMAN | Poly [ADP-ribose] polymerase 1 OS=Homo sapiens OX=9606 GN=PARP1 PE=1 SV=4 | 113 |
| sp\|Q92499\|DDX1_HUMAN | ATP-dependent RNA helicase DDX1 OS=Homo sapiens OX=9606 GN=DDX1 PE=1 SV=2 | 82 |
| sp\|Q00610\|CLH1_HUMAN | Clathrin heavy chain 1 OS=Homo sapiens OX=9606 GN=CLTC PE=1 SV=5 | 192 |
| tr\|A0A087WVQ6\|A0A087WVQ6_HUMAN | Clathrin heavy chain OS=Homo sapiens OX=9606 GN=CLTC PE=1 SV=1 | 192 |
| sp\|P27708\|PYR1_HUMAN | CAD protein OS=Homo sapiens OX=9606 GN=CAD PE=1 SV=3 | 243 |
| sp\|P19338\|NUCL_HUMAN | Nucleolin OS=Homo sapiens OX=9606 GN=NCL PE=1 SV=3 | 77 |
| sp\|Q9P2I0\|CPSF2_HUMAN | Cleavage and polyadenylation specificity factor subunit 2 OS=Homo sapiens OX=9606 GN=CPSF2 PE=1 SV=2 | 88 |
| sp\|P11142\|HSP7C_HUMAN | Heat shock cognate 71 kDa protein OS=Homo sapiens OX=9606 GN=HSPA8 PE=1 SV=1 | 71 |
| sp\|Q9Y3I0\|RTCB_HUMAN | tRNA-splicing ligase RtcB homolog OS=Homo sapiens OX=9606 GN=RTCB PE=1 SV=1 | 55 |
| sp\|P49916\|DNLI3_HUMAN | DNA ligase 3 OS=Homo sapiens OX=9606 GN=LIG3 PE=1 SV=2 | 113 |
| sp\|P06748\|NPM_HUMAN | Nucleophosmin OS=Homo sapiens OX=9606 GN=NPM1 PE=1 SV=2 | 33 |
| sp\|P13639\|EF2_HUMAN | Elongation factor 2 OS=Homo sapiens OX=9606 GN=EEF2 PE=1 SV=4 | 95 |
| sp\|Q9UKF6\|CPSF3_HUMAN | Cleavage and polyadenylation specificity factor subunit 3 OS=Homo sapiens OX=9606 GN=CPSF3 PE=1 SV=1 | 77 |
| sp\|O75533\|SF3B1_HUMAN | Splicing factor 3B subunit 1 OS=Homo sapiens OX=9606 GN=SF3B1 PE=1 SV=3 | 146 |
| sp\|P08238\|HS90B_HUMAN | Heat shock protein HSP 90-beta OS=Homo sapiens OX=9606 GN=HSP90AB1 PE=1 SV=4 | 83 |
| tr\|C9J9K3\|C9J9K3_HUMAN | 40S ribosomal protein SA (Fragment) OS=Homo sapiens OX=9606 GN=RPSA PE=1 SV=8 | 29 |
| sp\|Q15437\|SC23B_HUMAN | Protein transport protein Sec23B OS=Homo sapiens OX=9606 GN=SEC23B PE=1 SV=2 | 86 |
| sp\|Q7KZF4\|SND1_HUMAN | Staphylococcal nuclease domain-containing protein 1 OS=Homo sapiens OX=9606 GN=SND1 PE=1 SV=1 | 102 |
| sp\|Q6UN15\|FIP1_HUMAN | Pre-mRNA 3'-end-processing factor FIP1 OS=Homo sapiens OX=9606 GN=FIP1L1 PE=1 SV=1 | 67 |
| sp\|P26196\|DDX6_HUMAN | Probable ATP-dependent RNA helicase DDX6 OS=Homo sapiens OX=9606 GN=DDX6 PE=1 SV=2 | 54 |
| sp\|P53992\|SC24C_HUMAN | Protein transport protein Sec24C OS=Homo sapiens OX=9606 GN=SEC24C PE=1 SV=3 | 118 |
| sp\|P35573\|GDE_HUMAN | Glycogen debranching enzyme OS=Homo sapiens OX=9606 GN=AGL PE=1 SV=3 | 175 |
| sp\|P67809\|YBOX1_HUMAN | Nuclease-sensitive element-binding protein 1 OS=Homo sapiens OX=9606 GN=YBX1 PE=1 SV=3 | 36 |
| sp\|Q7Z406\|MYH14_HUMAN | Myosin-14 OS=Homo sapiens OX=9606 GN=MYH14 PE=1 SV=2 | 228 |
| tr\|E7EX17\|E7EX17_HUMAN | Eukaryotic translation initiation factor 4B OS=Homo sapiens OX=9606 GN=EIF4B PE=1 SV=1 | 70 |
| sp\|O75131\|CPNE3_HUMAN | Copine-3 OS=Homo sapiens OX=9606 GN=CPNE3 PE=1 SV=1 | 60 |
| sp\|Q13435\|SF3B2_HUMAN | Splicing factor 3B subunit 2 OS=Homo sapiens OX=9606 GN=SF3B2 PE=1 SV=2 | 100 |
| sp\|P23396\|RS3_HUMAN | 40S ribosomal protein S3 OS=Homo sapiens OX=9606 GN=RPS3 PE=1 SV=2 | 27 |
| sp\|Q32P28\|P3H1_HUMAN | Prolyl 3-hydroxylase 1 OS=Homo sapiens OX=9606 GN=P3H1 PE=1 SV=2 | 83 |
| sp\|P51659\|DHB4_HUMAN | Peroxisomal multifunctional enzyme type 2 OS=Homo sapiens OX=9606 GN=HSD17B4 PE=1 SV=3 | 80 |
| sp\|Q8NC51\|PAIRB_HUMAN | Plasminogen activator inhibitor 1 RNA-binding protein OS=Homo sapiens OX=9606 GN=SERBP1 PE=1 SV=2 | 45 |
| sp\|P08174\|DAF_HUMAN | Complement decay-accelerating factor OS=Homo sapiens OX=9606 GN=CD55 PE=1 SV=4 | 41 |
| sp\|P63244\|RACK1_HUMAN | Receptor of activated protein C kinase 1 OS=Homo sapiens OX=9606 GN=RACK1 PE=1 SV=3 | 35 |
| sp\|Q9Y5X1\|SNX9_HUMAN | Sorting nexin-9 OS=Homo sapiens OX=9606 GN=SNX9 PE=1 SV=1 | 67 |
| sp\|Q6WCQ1\|MPRIP_HUMAN | Myosin phosphatase Rho-interacting protein OS=Homo sapiens OX=9606 GN=MPRIP PE=1 SV=3 | 117 |
| sp\|Q96AE4\|FUBP1_HUMAN | Far upstream element-binding protein 1 OS=Homo sapiens OX=9606 GN=FUBP1 PE=1 SV=3 | 68 |
| sp\|Q96I25\|SPF45_HUMAN | Splicing factor 45 OS=Homo sapiens OX=9606 GN=RBM17 PE=1 SV=1 | 45 |
| tr\|A0A0A0MS51\|A0A0A0MS51_HUMAN | Gelsolin OS=Homo sapiens OX=9606 GN=GSN PE=1 SV=1 | 83 |
| sp\|Q9ULV4\|COR1C_HUMAN | Coronin-1C OS=Homo sapiens OX=9606 GN=CORO1C PE=1 SV=1 | 53 |
| sp\|Q92614\|MY18A_HUMAN | Unconventional myosin-XVIIIa OS=Homo sapiens OX=9606 GN=MYO18A PE=1 SV=3 | 233 |
| sp\|Q9H0M0\|WWP1_HUMAN | NEDD4-like E3 ubiquitin-protein ligase WWP1 OS=Homo sapiens OX=9606 GN=WWP1 PE=1 SV=1 | 105 |
| sp\|Q92900\|RENT1_HUMAN | Regulator of nonsense transcripts 1 OS=Homo sapiens OX=9606 GN=UPF1 PE=1 SV=2 | 124 |
| sp\|P14923\|PLAK_HUMAN | Junction plakoglobin OS=Homo sapiens OX=9606 GN=JUP PE=1 SV=3 | 82 |
| tr\|A0A2R8YFS5\|A0A2R8YFS5_HUMAN | ATP-dependent RNA helicase DDX3X OS=Homo sapiens OX=9606 GN=DDX3X PE=1 SV=1 | 73 |
| sp\|O00571\|DDX3X_HUMAN | ATP-dependent RNA helicase DDX3X OS=Homo sapiens OX=9606 GN=DDX3X PE=1 SV=3 | 73 |
| sp\|P05388\|RLA0_HUMAN | 60S acidic ribosomal protein P0 OS=Homo sapiens OX=9606 GN=RPLP0 PE=1 SV=1 | 34 |
| sp\|Q9NVC6\|MED17_HUMAN | Mediator of RNA polymerase II transcription subunit 17 OS=Homo sapiens OX=9606 GN=MED17 PE=1 SV=2 | 73 |
| sp\|A5YKK6\|CNOT1_HUMAN | CCR4-NOT transcription complex subunit 1 OS=Homo sapiens OX=9606 GN=CNOT1 PE=1 SV=2 | 267 |
| tr\|H3BNC9\|H3BNC9_HUMAN | Uncharacterized protein OS=Homo sapiens OX=9606 PE=3 SV=2 | 65 |
| sp\|Q15459\|SF3A1_HUMAN | Splicing factor 3A subunit 1 OS=Homo sapiens OX=9606 GN=SF3A1 PE=1 SV=1 | 89 |
| sp\|P08708\|RS17_HUMAN | 40S ribosomal protein S17 OS=Homo sapiens OX=9606 GN=RPS17 PE=1 SV=2 | 16 |
| sp\|P12532\|KCRU_HUMAN | Creatine kinase U-type mitochondrial OS=Homo sapiens OX=9606 GN=CKMT1A PE=1 SV=1 | 47 |
| sp\|O75448\|MED24_HUMAN | Mediator of RNA polymerase II transcription subunit 24 OS=Homo sapiens OX=9606 GN=MED24 PE=1 SV=1 | 110 |
| sp\|O43491\|E41L2_HUMAN | Band 4.1-like protein 2 OS=Homo sapiens OX=9606 GN=EPB41L2 PE=1 SV=1 | 113 |
| sp\|P27824\|CALX_HUMAN | Calnexin OS=Homo sapiens OX=9606 GN=CANX PE=1 SV=2 | 68 |
| sp\|O60244\|MED14_HUMAN | Mediator of RNA polymerase II transcription subunit 14 OS=Homo sapiens OX=9606 GN=MED14 PE=1 SV=2 | 161 |

| **Supplementary Table 2. primer sequences for RT-PCR** | |
| --- | --- |
| WWP1-F | TGCTTCACCAAGGTCTGATACT |
| WWP1-R | GCTGTTCCGAACCAGTTCTTTT |
| MUC1-F | TGTCAGTGCCGCCGAAAGAA |
| MUC1-R | CTACAAGTTGGCAGAAGTGG |
| GAPDH-F | ATGTTCGTCATGGGTGTGAA |
| GAPDH-R | GGTGCTAAGCAGTTGGTGGT |

**References**

1. Lv, Y. et al. Erlotinib overcomes paclitaxel-resistant cancer stem cells by blocking the EGFR-CREB/GRbeta-IL-6 axis in MUC1-positive cervical cancer. *Oncogenesis*. **8**, 70 (2019).

2. Gao, J. et al. Integrative analysis of complex cancer genomics and clinical profiles using the cBioPortal. *Sci. Signal.* **6**, l1 (2013).

3. Cerami, E. et al. The cBio cancer genomics portal: an open platform for exploring multidimensional cancer genomics data. *Cancer Discov.* **2**, 401-404 (2012).
